# Effects of Individual or Combined Deficiency of Phosphorous and Zinc on Phenotypic, Nutrient Uptake, and Molecular Responses of Finger Millet (*Eleusine coracana*): A Nutri-Rich Cereal Crop

**DOI:** 10.3390/plants12193378

**Published:** 2023-09-25

**Authors:** Theivanayagam Maharajan, Thumadath Palayullaparambil Ajeesh Krishna, Jayabalan Shilpha, Stanislaus Antony Ceasar

**Affiliations:** 1 Division of Plant Molecular Biology and Biotechnology, Department of Biosciences, Rajagiri College of Social Sciences, Cochin 683104, India; susirajan143@gmail.com (T.M.); ajeeshkrishnatp@gmail.com (T.P.A.K.); 2 Department of Horticulture, Institute of Agriculture and Life Science, Gyeongsang National University, Jinju 52828, Republic of Korea; shilphajayabalan@gmail.com

**Keywords:** finger millet, gene expression, phosphorus (P), phosphate transporter 1 (PHT1), zinc (Zn), zinc-regulated, iron-regulated transporter-like proteins (ZIP)

## Abstract

Deficiencies of either phosphorus (P) or zinc (Zn) or both are one of the major abiotic constraints influencing agricultural production. Research on the effects of individual or combined P and Zn deficiency is limited in cereals. This study reports the effects of the individual or combined deficiency of inorganic phosphate (Pi) and Zn on the phenotypic, root hair modification, nutrient uptake, and molecular responses of finger millet (*Eleusine coracana*), a nutri-rich cereal crop. Finger millet seedlings were grown hydroponically under control (+Pi+Zn), individual Pi deficiency (−Pi), individual Zn deficiency (−Zn), and combined Pi and Zn deficiency (−Pi−Zn) conditions for 30 days to find the phenotypic, root hair modification, nutrient uptake, and molecular responses. Compared to the individual −Zn condition, the individual −Pi condition had more of an effect in terms of biomass reduction. The combined −Pi−Zn condition increased the root hair length and density compared to the other three conditions. The individual −Zn condition increased the Pi uptake, while the individual −Pi condition favored the Zn uptake. *EcZIP2* was highly upregulated in shoot tissues under the individual −Zn condition, and *EcPHT1*;*2* was highly expressed in root tissues under the individual −Pi condition. This is the first study to report the effects of the individual or combined deficiency of Pi and Zn in finger millet and may lead to future studies to better manage P and Zn deficiency.

## 1. Introduction

Millets are cultivated worldwide, particularly in Asian and African countries. Crops such as rice (*Oryza sativa*), wheat (*Triticum aestivum*), and maize (*Zea mays*) supply all the necessary nutrients, vitamins, and energy for human survival in tropical and sub-tropical regions [1]. Three unique names are frequently used to refer to millet: a “nutri-ceral” due to its nutrient-dense seeds; a “poor man’s cereal” as it improves the life and health of the poor; and a “climate-resilient cereal” since it can survive in adverse climatic conditions in tropical and sub-tropical regions [2]. Considering the importance of millets for human life, the United Nations (UN) declared 2023 as the International Year of Millets. Each millet has some specific nutrients and health benefits. Among the seven millets, finger millet (*Eleusine coracana*) has higher amounts of calcium (Ca) and is called a “Ca-rich” cereal [3]. Millets are considered climate-hardy crops since they are somewhat tolerant to adverse climates compared to major cereals. However, they cannot survive and do not produce a sufficient yield when grown under low nutrients, as proven by previous reports [4,5]. The growth and plant biomass of over 50 finger millet genotypes were reduced under an individual inorganic phosphate (Pi) deficiency (−Pi) condition [6]. The growth, biomass, yield, total phosphorus (TP), and Pi contents were significantly reduced in seven millets under individual −Pi condition [7,8]. The growth, biomass, flower morphology, and yield were reduced in more than 30 foxtail millet (*Setaria italica*) genotypes grown in individual −Pi soil for two seasons [9]. In another study, Pi and TP contents were reduced in the above- (shoot) and below-ground (root) tissues of more than ten foxtail millet genotypes grown under individual −Pi condition [10]. The individual zinc (Zn) deficiency (−Zn) condition also influenced the growth, biomass, and yield of seven millets [11]. In the same study, the Zn content was reduced in 30-day-old seedlings of the shoot and root tissues of seven millets under the individual −Zn condition. Hence, P and Zn deficiencies are major abiotic stresses for millet growth and yield. Only a few reports are available on the responses of millets under nutrient deficiencies. The signaling and transport mechanism of nutrients in finger millet has not yet been reported.

Plants can produce a good yield when grown with sufficient nutrients. Each macro- and micronutrient has a specific role in plants. Phosphorus (P) is one of the most important macronutrients for plants [12]. It is particularly involved in respiration and photosynthesis from when plants are seedlings to grain formation and maturity [13,14]. Like other micronutrients, Zn is also one of the essential micronutrients for plants’ life cycles [15]. It is a key component of many proteins and enzymes. It contributes to several important plant functions, such as growth hormone production and internode elongation [16]. Pi and Zn are important macro- and micronutrients, respectively, required for normal plant growth. Plants absorb these two elements from soil solutions by the root system.

Both P and Zn levels are low in agricultural land worldwide, which negatively affects plant metabolism and photosynthesis. Around 80% and over 50% of agricultural land do not contain sufficient P and Zn, respectively [13,15]. As a result, deficiencies of either Pi or Zn or both have become a global concern in terms of food availability and nutrient deficiency in recent years. Some considerable efforts have already been made by researchers to minimize these critical situations. The effects of individual or combined deficiency of Pi and Zn on Pi and Zn uptake have been reported in various plants (Table S1). The P concentration was higher in barley (*Hordeum vulgare*) shoots when plants were grown under the individual −Zn condition [17]. In contrast, under individual −Pi condition, the P concentration increased significantly in the grains and straws of wheat and maize [18]. The leaf Zn concentration was reduced in dwarf beans (*Phaseolus vulgaris*) grown under the individual −Zn condition [19]. In Arabidopsis (*Arabidopsis thaliana*), the P content was increased in both the shoot and root tissues of plants grown under the individual −Zn condition and vice versa [20]. Thus, the individual deficiency of Pi or Zn is a major abiotic stress for cereals, including millets. All these reports have revealed that higher concentrations of either Pi or Zn in soil lead to a deficiency of the opposite nutrients in plants due to the antagonistic effect between these two nutrients. In addition, lower concentrations of either Pi or Zn in soil pave the way for the over-accumulation of Zn or P, respectively. Apart from the individual −Pi and individual −Zn conditions, the excess of both (++Pi and ++Zn) reduced all phenotype traits in plants, including millets [8,11]. In general, the uptake, availability, and utilization of other nutrients are affected by the excess of both (++Pi and ++Zn) in growing media [21]. It has been reported that the individual ++Pi condition reduces the uptake and utilization of various nutrients (Zn, potassium (K), and iron (Fe)), and the individual ++Zn condition reduces the uptake and utilization of Pi and Fe in cereals [22]. Thus, studying the effects of the individual or combined deficiency of Pi and Zn in finger millet is important. This is the first study to investigate the effects of the individual or combined deficiency of Pi and Zn in millet.

Multiple phosphate transporter 1 (PHT1) and zinc-regulated, Fe-regulated trans-porter-like protein (ZIP) family genes are involved in the uptake, translocation, and re-mobilization of Pi and Zn, respectively [12,16]. *PHT1* family genes were induced in various tissues of a few plants under the individual −Zn condition. In Arabidopsis, the individual −Zn condition upregulated the expression of the *AtPHT1*;*1* gene in shoot tissues with a higher uptake of Pi over Zn compared to that of +Pi+Zn (control) [23]. In another study, two high-affinity *PHT1* family transporter genes (*HvPHT1*;*1* and *HvPHT1*;*2*) were found to be expressed in the root tissue of barley grown under the individual −Zn condition [17]. The expression level of these two genes is closely linked to high TP and reduced Zn content in its shoot and root tissues. In Arabidopsis, nine *PHT1* (*AtPHT1*;*1* to *AtPHT1*;*9*) genes were expressed in shoots and roots under the individual −Zn condition with a higher uptake of Pi over Zn [20]. These studies revealed that *PHT1* family transporter genes are upregulated when plants are grown under the individual −Zn condition, which increases the Pi level and downregulation of *ZIP* genes with a lower uptake of Zn in plant tissues. In contrast, when plants are grown under the individual −Pi condition, they accumulate more Zn in their shoot and root tissues or any other aerial parts due to the downregulation of *PHT1* family genes. All previous studies have shown that the individual deficiency of Pi or Zn has antagonistic effects on plant growth.

This study aims to analyze the phenotypic modification, root hair modification, nutrient update, and expression analysis of *PHT1* and *ZIP* family transporters in shoot and root tissues of finger millet under the individual or combined deficiency of Pi and Zn. The effects of the individual or combined deficiency of Pi and Zn at physiological, biochemical, and molecular levels have not yet been reported in any millet, including finger millet. This study will help millet researchers understand how the individual or combined deficiency of Pi and Zn influences the phenotype, root hair modification, and Pi and Zn uptake at the seedling stage.

## 2. Results and Discussion

### 2.1. Phenotypic and Biochemical Changes under Individual or Combined Deficiency of Pi and Zn

#### 2.1.1. Individual or Combined Deficiency of Pi and Zn Influenced Seedling Growth and Biomass

**Figure 1:**
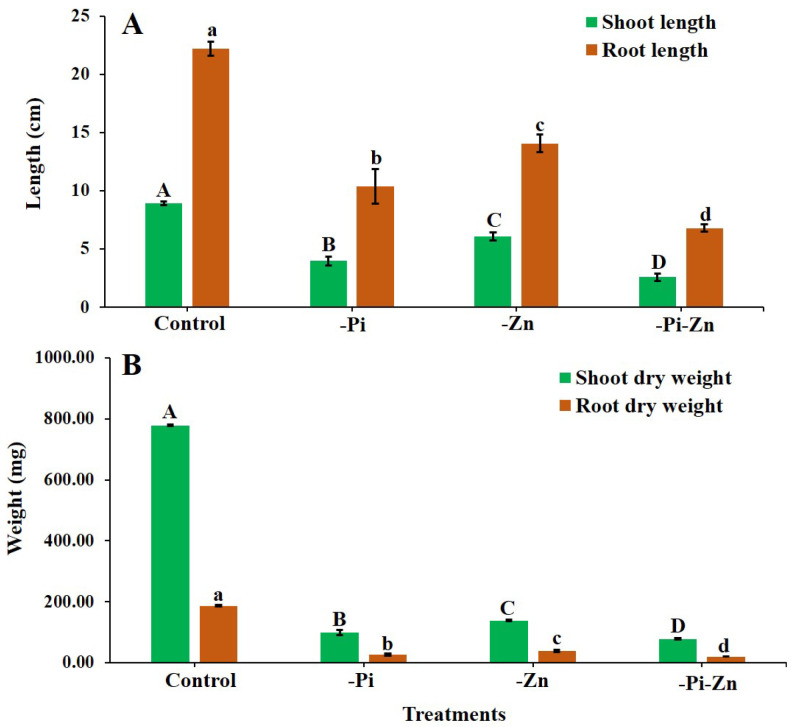
Effects of individual or combined deficiency of Pi and Zn on growth and biomass of finger millet. The finger millet plants grown in hydroponics under control (+Pi+Zn), individual Pi deficiency (−Pi), individual Zn deficiency (−Zn), and combined Pi and Zn deficiency (−Pi−Zn) conditions. Shoot and root length (**A**) and shoot and root dry weight (**B**) were measured from 30-day-old seedlings of finger millet. Significant differences among treatments were indicated in upper case (A,B,C,D) for shoot length and shoot dry weight, and in lower case (a,b,c,d) for root length and root dry weight.

The individual or combined deficiency of Pi and Zn influenced the phenotypic traits of finger millet. Finger millet seedings grown under the individual or combined deficiency of Pi and Zn conditions showed varied responses for shoot length (SL), primary root length (PRL), shoot dry weight (SDW), and root dry weight (RDW). As expected, the highest reduction in the SL (71.3%), PRL (69.3%), SDW (89.9%), and RDW (89.0%) was found when the finger millet was grown under the combined −Pi−Zn conditions compared to that of the +Pi+Zn, individual −Pi, and individual −Zn conditions (Figure 1A,B). The individual −Pi condition also resulted in a higher reduction in the SL (55.6%), PRL (53.6%), SDW (87.3%), and RDW (85.5%) compared to that of the individual −Zn and +Pi+Zn conditions. However, the individual −Zn condition showed a smaller effect on the reduction in the SL (32.1%), PRL (37.0%), SDW (82.1%), and RDW (79.5%) than the +Pi+Zn condition (Figure 1A,B).

Interestingly, plant growth and biomass significantly increased under the individual −Zn condition compared to that under the individual −Pi condition. The finger millet seedlings could better tolerate the individual −Zn condition compared to the individual −Pi condition based on this study. Similarly, in a previous report on wheat, compared to the individual −Pi condition, the individual −Zn condition had a small effect on the SDW and RDW [24]. In maize, the SDW and RDW were not significantly affected under the individual −Zn condition compared to the individual −Pi condition [25,26]. A similar trend was also found in rice [27,28], okra [29], and barley [30,31]. Other previous studies have also reported that the individual or combined deficiency of Pi and Zn influenced the SL, PRL, SDW, and RDW in maize [32], barley [33], rice [34], wheat [35], soybean (*Glycine max*) [36], foxtail millet [37], and other millets [8,11]. Based on the phenotypic traits and our biomass analysis, we conclude that the combined −Pi−Zn condition had the maximum adverse effect on the finger millet seedlings compared to the individual −Pi and individual −Zn conditions. Additionally, compared to the individual −Zn condition, the growth and biomass of finger millet were significantly reduced under the individual −Pi condition. Since P is a major nutrient, the individual −Pi condition seems to have more of an impact on finger millet compared to that of the individual −Zn condition. Hence, growing millet under sufficient Pi and Zn is essential for healthy plant life. The effect of the individual or combined deficiency of Pi and Zn has not yet been studied extensively, and no information is available on cereals, including millets. Hence, this study will inform the scientific community about the phenotypic effects of finger millet under the individual or combined deficiency of Pi and Zn. This study will raise awareness among plant nutrient researchers to initiate more research on Pi and Zn in millets in the future.

#### 2.1.2. Individual or Combined Deficiency of Pi and Zn Influenced the Root Hair Modification

**Figure 2:**
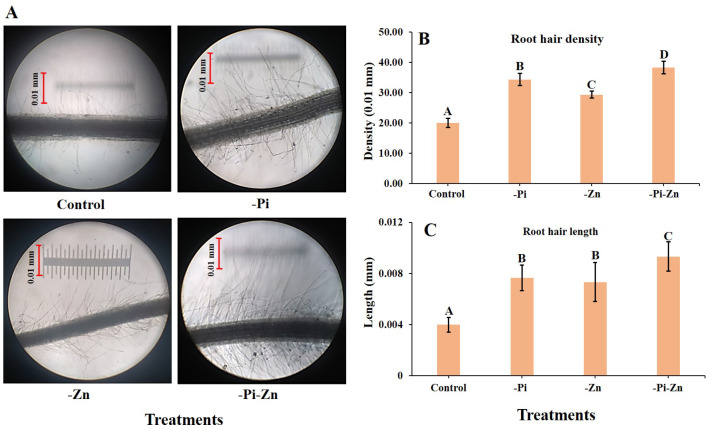
Effects of individual or combined deficiency of Pi and Zn on root hair modification of finger millet. The finger millet plants grown in hydroponics under control (+Pi+Zn), individual Zn deficiency (−Zn), individual Pi deficiency (−Pi), and combined Pi and Zn deficiency (−Pi−Zn) conditions. Images of root hair (**A**) were obtained from 30-day-old seedlings of finger millet using 10× magnification in a microscope (Leitz Wetzlar Germany 513558 (Model no. Laborlux 12)). The root hair density (**B**) and root hair length (**C**) were counted using the ImageJ V.1.8.0 scientific software (http://imagej.net/); accessed on 24 March 2023. Significant differences among treatments were indicated in upper case (A,B,C,D) letters for root hair length and root hair density separately.

Genetic and environmental factors influence the root hair growth of plants. Their growth and density increased under drought and nutrient deficiency conditions (including individual −Pi and individual −Zn condition). Root hair modification may be an important strategy to improve Pi and Zn acquisition when the Pi and Zn availability is low in the soil. The individual or combined deficiency of Pi and Zn altered the root hair of finger millet seedlings. The captured root hair images of finger millet under the individual or combined deficiency of Pi and Zn are projected in Figure 2A. Interestingly, we have seen a significantly higher root hair density (RHD) and root hair length (RHL) under the combined −Pi−Zn condition compared to the +Pi+Zn condition, individual −Pi condition, and individual −Zn condition. The RHL increased by 55.5% and the RHD by 47.8% under the combined −Pi−Zn condition compared to those of the +Pi+Zn condition (Figure 2B,C).

The combined effects of the −Pi−Zn condition on root hair modification have not yet been reported in any cereal. Root hair induction is an adaptive mechanism by plants [38] wherein they try to find and uptake more nutrients by root hair induction under individual −Pi condition [39] and individual −Zn condition [40]. Compared to the +Pi+Zn condition, the individual −Pi condition showed a 44% increase in RHL and 42% increase in RHD, whereas the individual −Zn condition showed a 42.8% increase in RHL and 31.8% increase in RHD. Thus, the individual −Pi condition has the maximum effect, inducing a higher RHL and RHD than the individual −Zn condition in finger millet seedlings. In previous reports, a higher RHD and RHL were seen under individual −Pi condition in Arabidopsis [38], maize [41], barley [42], finger millet [8], and wheat [43] and individual −Zn condition in maize [44] and barley [33]. Very recently, the effects of individual −Zn condition on root hair modification have been reported in seven millets [11]. All seven millets produced a higher RHL and RHD under the individual −Zn condition [11] compared to the +Pi+Zn condition. The present study revealed that a higher RHL and RHD were produced under the combined −Pi−Zn condition compared to those of the individual −Pi and individual −Zn condition. Phenotypic modification, especially of RHD and RHL, is a key trait required for breeding and crop improvements to overcome the low-nutrient stress in agriculture fields [45]. Thus, further study on this trait in finger and other millets could help improve the nutrient uptake under combined −Pi−Zn soils. Several genes have been reported to regulate root hair initiation and elongation under both individual −Pi and individual −Zn conditions [11]. Hence, identifying and characterizing root-hair-responsive genes from the finger millet genome will help us to understand and improve root hair growth and nutrient update under individual −Pi, individual −Zn, and combined −Pi−Zn conditions.

#### 2.1.3. Individual or Combined Deficiency of Pi and Zn Influenced the Pi and Zn Content

**Figure 3:**
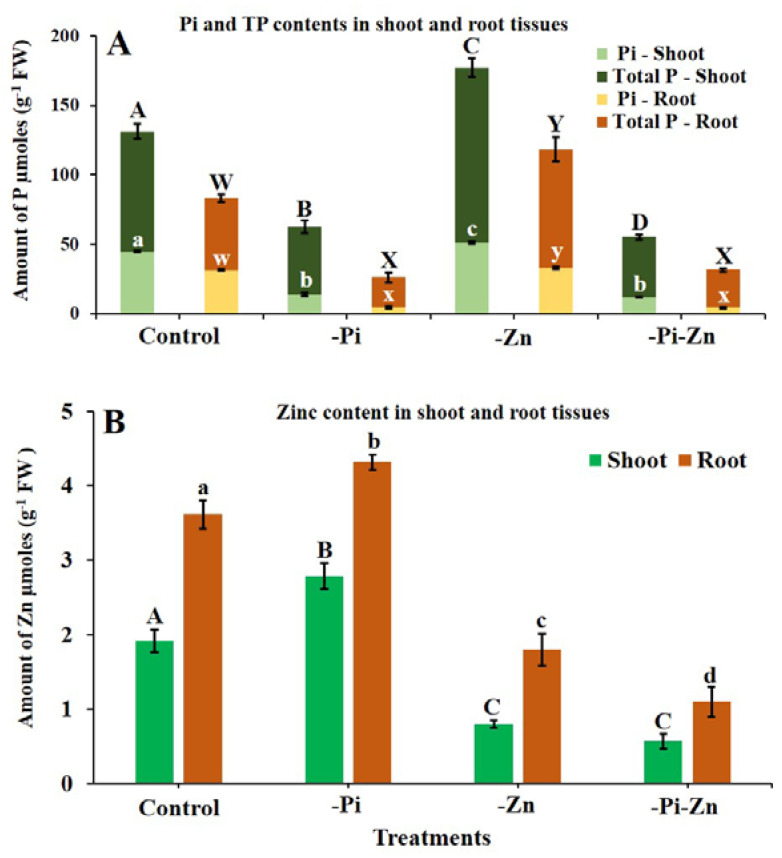
Analysis of Pi, TP, and Zn contents in shoot and root tissues of finger millet under individual or combined deficiency of Pi and Zn. The Pi, TP contents (**A**), and Zn contents (**B**) were analyzed in finger millet under control (+Pi+Zn), individual Zn deficiency (−Zn), individual Pi deficiency (−Pi), and combined Pi and Zn deficiency (−Pi−Zn) conditions. Significant differences among treatment were indicated in upper case (A,B,C,D) for shoot Pi contents and shoot Zn contents and in lower case (a,b,c,d) for shoot TP content and root Zn contents. Significant difference among treatments were indicated in upper case (W,X,Y) for root Pi content and in lower case (w,x,y) for root TP content.

In the present study, the contents of Pi, TP, and Zn were examined in finger millet shoot and root tissues under the individual or combined deficiency of Pi and Zn. Under the individual −Pi condition, as predicted, the contents of TP and Pi were significantly reduced (>43%) in shoots and roots compared to those under the +Pi+Zn condition (Figure 3A). In the present study, the Pi contents were reduced by >70% in root and shoot tissues under the individual −Pi condition compared to those under the +Pi+Zn condition in rice [46], by >40% in foxtail millet [9,10,37], by >10% in maize [47], and by >60% in seven millets [8]. Contrastingly, the contents of Zn significantly increased in the shoot (31.2%) and root (16.2%) tissues of finger millet under the same individual −Pi condition (Figure 3B). Concurrent with this study, the Zn content was higher (>20%) in the shoot and root tissues of wheat [48] under the individual −Pi condition. The individual −Pi condition increased the Zn content by >25% in the root tissues of cotton [49]. The individual −Pi condition also promoted Zn uptake in the shoot and root tissues of rice [28,50], maize [26,51], and sorghum [52]. Under the individual −Zn condition, the contents of Pi and TP in the shoot and root tissues were increased by >20% in finger millet compared to the +Pi+Zn condition (Figure 3A). As expected, the contents of Zn were reduced by >50% in both the shoot and root tissues under the individual −Zn condition in the present study. A very recent study also revealed that the contents of Zn were reduced by >40% in the shoot and root tissues of seven millets under the individual −Zn condition [11]. In maize, the Zn content decreased by >50% in the shoot tissue, and the TP content increased by >40% in shoot and root tissues under the individual −Zn condition [53]. In past studies, the individual −Zn condition favored the Pi uptake and reduced the Zn acquisition in maize [54], Arabidopsis [55], rice [50,56], sorghum [52,57], and dwarf bean [19]. It has been well documented in other plants that Pi and Zn have antagonistic effects on the uptake. As a result, the individual −Pi condition promoted Zn uptake, and the individual −Zn condition favored Pi accumulation. As expected, the inferior responses of the Zn and Pi uptake were seen under the combined −Pi−Zn condition. The Pi contents were significantly reduced in the shoots (73.5%) and roots (85.4%), and the Zn contents were reduced by 70% in the shoots and roots under the combined −Pi−Zn condition compared to those under the +Pi+Zn condition. The Zn and P contents were reduced by >50% in wheat under the combined −Pi−Zn condition compared to the +Pi+Zn condition [24]. In another experiment, both the Zn and Pi contents decreased (>40%) in root tissues when wheat was grown under the combined −Pi−Zn condition [58]. As with other cereals, the Pi and Zn contents were reduced in finger millet under the individual −Pi condition and individual −Zn condition, respectively. Apart from this, the contents of TP and Pi were reduced by >70% in the roots and shoots when finger millet was grown under the combined −Pi−Zn condition compared to the +Pi+Zn condition in this study. Additionally, the higher TP and Pi contents of the shoots and roots of millets under the +Pi+Zn condition lead to increased biomass accumulation in the shoots and roots. Like other plants, the deficiency of both P and Zn had a profound effect on finger millet compared to that of the deficiency of either Pi or Zn alone.

Further molecular studies focusing on the genes involved in the regulation of these nutrients in finger millet could help dissect any specific gene or network involved in low-Zn or -Pi tolerance mechanism in millets. In Arabidopsis, the *Lyso-PhosphatidylCholine AcylTransferase 1* (*LPCAT1*) gene increases the fatty acyl composition of PhosphatidylCholine, which in turn regulates *AtPHT1*;*1* gene expression, resulting in Pi accumulation in shoots under the individual −Zn condition [59]. Hence, LPCAT1 plays an important role in controlling Pi homeostasis under the individual −Zn condition [59].

#### 2.1.4. Individual or Combined Deficiency of Pi and Zn Influenced the Expression of EcPHT1 and EcZIP Family Genes

**Figure 4:**
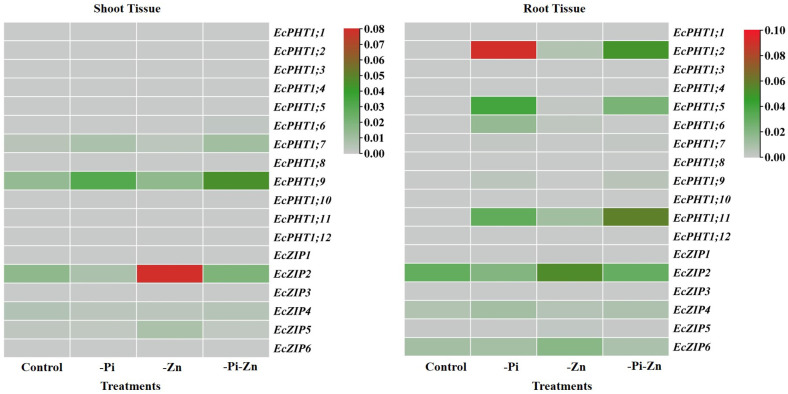
Analysis of expression of *EcPHT1* and *EcZIP* family transporter genes in shoot and root tissues of finger millet under individual or combined deficiency of Pi and Zn. Expression of 12 *EcPHT1* and six *EcZIP* family transporter genes were analyzed in 30-day-old seedlings of the shoot and root tissues of finger millet under control (+Pi+Zn), individual Zn deficiency (−Zn), individual Pi deficiency (−Pi), and combined Pi and Zn deficiency (−Pi−Zn) conditions.

The individual or combined deficiency of Pi and Zn revealed that these two nutrients had antagonistic effects on the uptake of each other in finger millet, which is similar to those reported in other plants. Thus, we were interested in investigating the expression of the *PHT1* and *ZIP* family genes, which are primarily involved in Pi [12] and Zn [16,60,61] transport, respectively. In the present study, 30-day-old shoot and root tissues were used to analyze the expression pattern of the 12 *EcPHT1* and 6 *EcZIP* family genes under the individual or combined deficiency of Pi and Zn (Figure 4 left and right. The melt curve of each *EcPHT1* and *EcZIP* is provided in Figure S1. Among the 18 genes, four genes (*EcPHT1*;*7*, *EcPHT1*;*9*, *EcZIP2*, and *EcZIP4*) were found to be expressed in shoot tissues under all conditions (Figure 4 left. Among the *ZIP* family genes, *EcZIP2* was highly expressed in shoot tissues under the individual −Zn condition. The concentration of Zn in the shoot and root tissues was reduced by >50% under the individual −Zn condition than that under the +Pi+Zn condition. It clearly demonstrates that the individual −Zn condition induced the *EcZIP2* expression in finger millet seedlings. This is a common response of plants for the expression of specific *ZIP* family transporter gene(s) in shoot or root tissues in response to the individual −Zn condition. In Arabidopsis, *AtZIP9* has been considered as the marker gene for Zn deficiency in shoot tissues and induced under the individual −Zn condition [62]. Similarly, the *SbZIP2* transporter gene was highly expressed in the shoot tissues of sorghum under the individual −Zn condition [57]. *EcZIP2* was also slightly expressed in shoot tissues under the +Pi+Zn and combined −Pi−Zn conditions in finger millet. Under the individual −Zn condition, *ZmZIP2* was highly expressed in the various tissues (flag leaf and kernel) of maize [63]. Thus, the individual −Zn condition may induce *EcZIP2* in the flag leaf and developing spike of finger millet. Among *PHT1* family genes, *EcPHT1*;*9* was moderately expressed in shoot tissues under the individual −Pi condition and combined −Pi−Zn condition. *SbPHT1*;*9* was upregulated >two-fold in the leaves of sorghum under the individual −Pi condition [64]. In another study, the same *SiPHT1*;*9* was moderately expressed in the leave tissues of sorghum under the individual −Pi condition [65]. In soybean, *GmPHT1*;*9* was highly expressed in the stem and moderately expressed in the flower under the individual −Pi condition [66]. Based on the previous and present experiments, we assume that *EcPHT1*;*9* may be involved in the remobilization of Pi and can be expressed in developing spikes and other tissues during the maturation stage under the individual −Pi condition in finger millet.

In root tissue, among the *PHT1* family genes, the *EcPHT1*;*2* gene was highly expressed in the individual −Pi condition, and the same gene was moderately and slightly expressed in the combined −Pi−Zn and individual −Zn conditions, respectively (Figure 4 right). In most of the plants, the individual −Pi condition induced more *PHT1* family genes in the root tissues compared to the +Pi+Zn condition [14,15]. In previous studies, many *PHT1* family genes were reported to be induced in root tissues by the individual −Pi condition, including *AtPHT1*;*2* in Arabidopsis [67], *OsPHT1*;*2* in rice [68], and *TaPHT1*;*2* in wheat [69,70]. Interestingly, in our previous study, our gene expression analysis revealed that the *SiPHT1*;*2* gene is a constitutive one induced under all Pi conditions in foxtail millet [71], and the downregulation of this gene in plants had a great impact on Pi uptake [72]. Based on our previous results, we assume that *EcPHT1*;*2* might be a root-specific transporter under the individual −Pi condition in finger millet. A further characterization of the *EcPHT1*;*2* gene in yeast and in planta would help to dissect the definite role of this gene in finger millet. In the present study, *EcPHT1*;*5* and *EcPHT1*;*11* were moderately expressed in the root tissue of finger millet under the individual −Pi or combined −Pi−Zn conditions. *ZmPHT1*;*5* and *ZmPHT1*;*11* were highly induced by the individual −Pi condition in the root tissue of maize [73]. *TaPHT1*;*11* was highly expressed in the root tissue of wheat under the individual −Pi condition [70]. In our previous study, *SiPHT1*;*11* was moderately expressed in the root tissue of barnyard millet [8]. Further functional characterization would help to identify the specific roles of the *EcPHT1*;*5* and *EcPHT1*;*11* genes in finger millet. Among root-specific *ZIP* genes, the *EcZIP2* gene was found to be moderately expressed in root tissue under the individual −Zn condition compared to other conditions. This gene was induced by the individual −Zn condition in both the shoot and root tissues of finger millet in this study. The *AtZIP2* transporter was found as a high-affinity ZIP transporter in Arabidopsis under the individual −Zn condition [74]. In Arabidopsis and rice, the regulation of the *ZIP* and *PHT1* family genes by transcription factors has been well studied [28,30]. However, such studies are far from reaching millets, even though they are nutri-rich cereals [72]. Future high-resolution studies on Pi and Zn responses in finger millet would help to shed more light on the regulation and uptake of these nutrients under an individual or combined deficiency of Pi and Zn and other important nutrients.

## 3. Materials and Methods

### 3.1. Foundation Study to This Experiment

In our previous study, we identified more than five nutrient family transporters from the partially annotated genome sequences of finger millet, and we analyzed the protein features of all the identified nutrient transporters through in silico methods [72]. Among these, we have selected 12 *EcPHT1* and four *EcZIP* family transporters for this experiment as this study related to Pi and Zn transport. In the present experiment, we have additionally added two more ZIP family transporters (*EcZIP5* and *EcZIP6*) for gene expression analysis. Overall, 12 *EcPHT1* and six *EcZIP* family members were selected for gene expression analysis.

### 3.2. Plant Growth Experiments

We used the IE-2606 finger millet genotype collected from the International Crops Research Institute Semi-Arid Tropics (ICRISAT), Hyderabad, India for all experiments. Initially, the finger millet seeds were grown in perlite with a supply of essential macro- and micronutrients as per the previously published protocols [8]. One-week-old healthy seedlings were transferred into hydroponics tanks, which contained a sufficient amount of macro- and micronutrients, and were maintained for one week. After the establishment of seedlings, plants were transferred into hydroponics tanks having four different combinations of nutrients such as (a) sufficient Pi (500 µM) and sufficient Zn (1.0 µM) (+Pi+Zn) (control), (b) deficient Pi (10 µM) and sufficient Zn (1.0 µM) (individual Pi deficiency (−Pi) condition), (c) sufficient Pi (500 µM) and deficient Zn (0.05 µM) (individual Zn deficiency (−Zn) condition), and (d) deficient Pi (10 µM) and deficient Zn (0.05 µM) conditions (combined deficiency of P and Zn (−Pi−Zn)). The deficient (10 µM) and sufficient (500 µM) concentrations of Pi for finger millet growth were selected based on previous studies [8]. We have also identified the deficient (0.05 µM) and sufficient (1.0 µM) concentrations of Zn for finger millet growth and yield as per our recent report [11]. Nutrient solutions (with a pH range between 5.6 and 5.8) were renewed once in three days during the experiment. Three replicates (3 tanks for each condition) with each tank consisting of 9 plants were maintained.

### 3.3. Phenotypic and Biochemical Analysis

All phenotypic parameters, such as SL, PRL, SDW, RDW, RHL, and RHD, and biochemical traits (Pi, TP, and Zn contents in shoot and root) were measured in 30-day-old finger millet seedlings under individual −Pi, individual−Zn, combined −Pi−Zn, and control (+Pi+Zn) conditions based on the reported protocol [8,11]. The biochemical traits (Pi, TP, and Zn content in shoot and root tissues) were analyzed using the previously reported protocol [8,11]. Shoot and root tissues were collected from the same 30-day-old seedlings, and freshly harvested shoot and root tissues were stored at −80 °C and used for RNA isolation.

### 3.4. RNA Isolation and cDNA Synthesis

RNA was isolated from finger millet shoot and root tissues grown under individual −Pi, individual −Zn, combined −Pi−Zn, and +Pi+Zn conditions according to our previous protocol [8]. The quality and quantity of RNA were analyzed by agarose gel electrophoresis and Nanodrop spectrophotometer, respectively. cDNA was synthesized from the total RNA (500 ng) isolated from the shoot and root tissues using the QuantiTect Reverse transcription kit (Qiagen, Germany). Three biological replicates were used for each sample for RNA isolation and cDNA synthesis.

### 3.5. qRT-PCR Analysis

There were 18 gene-specific primers, including 12 *EcPHT1*s, 6 *EcZIP*s, and one constitutive gene (*EcEF1α*) of finger millet, were used for qRT-PCR analysis (Table 1). The expression of these genes was analyzed in finger millet shoots and roots grown under individual −Pi, individual −Zn, combined −Pi−Zn, and +Pi+Zn conditions based on our previously reported protocols [8,11]. *EcEF1α* was used to normalize the Ct values of each gene [8,11]. TBtools (https://bio.tools/tbtools; accessed on 21 April 2023) was used to differentiate the expression pattern of *EcPHT1* and *EcZIP* family genes in finger millet shoot and root tissues.

### 3.6. Statistical Analysis

Significant differences between traits were analyzed by one-way ANOVA using IBM SPSS statistics 25, and values were considered significant at *p* < 0.05.

## 4. Conclusions

In this study, finger millets accumulated more TP and Pi in their shoot and root tissues when the plants were grown under the individual −Zn condition and a higher content of Zn in both the shoot and root tissues grown under the individual −Pi condition in relation to those under the control condition. This caused the reduction in PRL, SL, SDW, and RDW under the individual −Zn and individual −Pi conditions. Apart from this, the SL, PRL, SDW, RDW, and contents of Pi, TP, and Zn were significantly reduced under the combined −Pi−Zn conditions compared to the other three conditions. Finger millet root hair traits (RHD and RHL) were increased (>20%) when they were grown under the individual −Pi condition, individual −Zn condition, or combined -Pi−Zn conditions compared to Pi+Zn; however, the impact of the individual −Pi was higher over that of the individual −Zn condition. *EcZIP2* was found to be highly expressed in shoot tissues under the individual −Zn condition, and *EcPHT1*;2 was highly expressed in root tissues under the individual −Pi condition compared to the other three treatments. Identifying key genes from this study would help improve finger millet growth and yield. The further functional characterization of identified key genes (particularly *EcZIP2* and *EcPHT1*;2) from this study would help us discover the exact role of these transporters. Further work is needed to dissect the transcription factors involved in the regulation of ZIP family genes under the individual −Pi condition, individual −Zn condition, or combined −Pi−Zn conditions in millet to find any novel interplay, as this has not been reported in any plants so far.

## Figures and Tables

**Table 1:** Details of *EcPHT1* and *EcZIP* primers used for qRT-PCR.

Gene Name	Forward Primer (5′–3′)	Reverse Primer (5′–3′)	Product Length (bp)	Tm (°C)
*EcPHT1;1*	GTTCTGCATCTCCCTTGTCTC	AATGAGCGTGAAGCCGTAGAC	202	60.1
*EcPHT1;2*	CCTGATCCTCATGGTGCTAT	AGAGGGTAGTCACCGCCTAT	129	58.9
*EcPHT1;3*	TTATCCACTATCCGCGACCA	CGAGATGATGAGCGTGACAA	130	58.1
*EcPHT1;4*	CAACAGCACCACCTTCGT	CCTAGCATGTTGGTGATGGA	195	58.9
*EcPHT1;5*	GCTGCGCTCACTTACTACTGG	CACCTGCTCATCGTCTTCC	141	59.5
*EcPHT1;6*	GCCATGCTCACCTACTACTGG	CGACTTGCAGAACCTTGGAC	115	60.6
*EcPHT1;7*	CCTCCACATGGACATCAGTG	CATGGCGATGCAGAAACTG	110	58.9
*EcPHT1;8*	ACGGTCTTCCAGTGCTCGT	GTTCACGGTCGCGTTCATC	117	59.3
*EcPHT1;9*	CAAGCGGATTTCGTGTGG	ACCTTTGACATGTCTGCTGCT	152	57.6
*EcPHT1;10*	CTTGACGAGCTGTACCACATC	ATATGGTTCTCCGCGGTCTT	200	59.3
*EcPHT1;11*	GGTGTACCTCGCAGTTTCC	TGTCACGCTCATCATCTCG	187	58.9
*EcPHT1;12*	GTGTACGGCTTCACTCTCATC	CGTCGCTGATAATGGATAGTCTC	153	60.1
*EcZIP1*	GACTCGCTCATGCTCACCTT	TGGTCCTTGTCTGCACCATC	159	59.3
*EcZIP2*	CCTGTGACACAACTACGGAAC	TCGTTGAGGACGAGCACAAG	158	59.8
*EcZIP3*	GGCAATGACAGTCCTCCTC	CCACCACCTGGTCCTTATT	127	57.8
*EcZIP4*	CACCTGGTGAAACGACAGTG	TGGAGATTGGGATTGTGTTC	110	57.5
*EcZIP5*	CTCCTCTGGTGAATTGATGAC	TGAGCAACATGGAGATGAGG	118	57.5
*EcZIP6*	GCGTCATCGTTGCAGTAGC	ACCACCAGCAGCAGACTC	110	58.6
*EcEF1-α*	CCTGGTGATAATGTGGGATTC	TAGCCATTGCCGATCTGTC	161	59.7

## Data Availability

All digital data (excel and word file) generated from this experiment are available at Department of Biosciences, Rajagiri College of Social Sciences. Researchers can collect the data through the corresponding author of this article.

## Supplementary Materials

The following supporting information can be downloaded at: https://www.mdpi.com/article/10.3390/plants12193378/s1, Figure S1: Images of melt curves of *EcEF-1α*, *EcPHT1* and *EcZIP* genes in Quantitative real-time RT-PCR (qRT-PCR) analysis for all samples; Table S1: Effect of individual or combined deficiency of Pi and Zn on Pi and Zn uptake in plants [17,20,23,30,53,58,73,74,75,76].
