# Supplementary material for: Effects of Individual or Combined Deficiency of Phosphorous and Zinc on Phenotypic, Nutrient Uptake, and Molecular Responses of Finger Millet (Eleusine coracana): A Nutri-Rich Cereal Crop
Source: Plants (Basel). 2023 Sep 25;12(19):3378. doi: 10.3390/plants12193378 (PMC10574462; doi:10.3390/plants12193378)
Supplement: Supplementary file 1 [file plants-12-03378-s001.zip › plants-2555557-supplementary.pdf]

Table S1. Effect of individual or combined deficiency of Pi and Zn on Pi and Zn uptake in plants

Plant Name	Botanical name	Condition	Effect on Zn/Pi uptake	Expression of *PHT1* genes	Reference
Arabidopsis	*Arabidopsis thaliana*	Individual -Zn condition	Increased Pi content in shoot	*AtPHT1;1* to *AtPHT1;9*	[20]
		Individual -Zn condition	Reduced Zn content and increased TP content in shoot	*AtPHT1;1*	[23]
Barley	*Hordeum Vulgare*	Individual -Zn condition	Increased TP content and reduced Zn content in shoot and root tissues	*HvPHT1;1* and *HvPHT1;2*	[17]
		Individual -Pi condition	Reduced, P content in shoot and root and increased Zn content in shoot	-	[73]
		Individual -Pi condition	Reduced TP content and increased Zn content in shoot and root	-	[30]
Maize	*Zea mays*	Individual -Zn condition	Reduced Zn content and increased total Pi contents in the shoot Increased Zn and TP content in root tissue	-	[53]
Wheat	*Triticum aestivum*	Individual -Pi condition	Reduced TP content and increased Zn in root, straw and grain	-	[58]
		Individual -Zn condition	Increased TP content in old leaves	-	[74]
		Combined deficiency of Pi and Zn	Reduced Zn and TP content in root, straw, grain	-	[58]
		Combined deficiency of Pi and Zn	Reduced Zn content in the shoot	-	[75]
lettuce	*Lactuca sativa*	Individual -Zn condition	Increased Pi content in root and shoot	-	[76]
		Combined deficiency of Pi and Zn	Reduced Zn and Pi content in shoot and root		

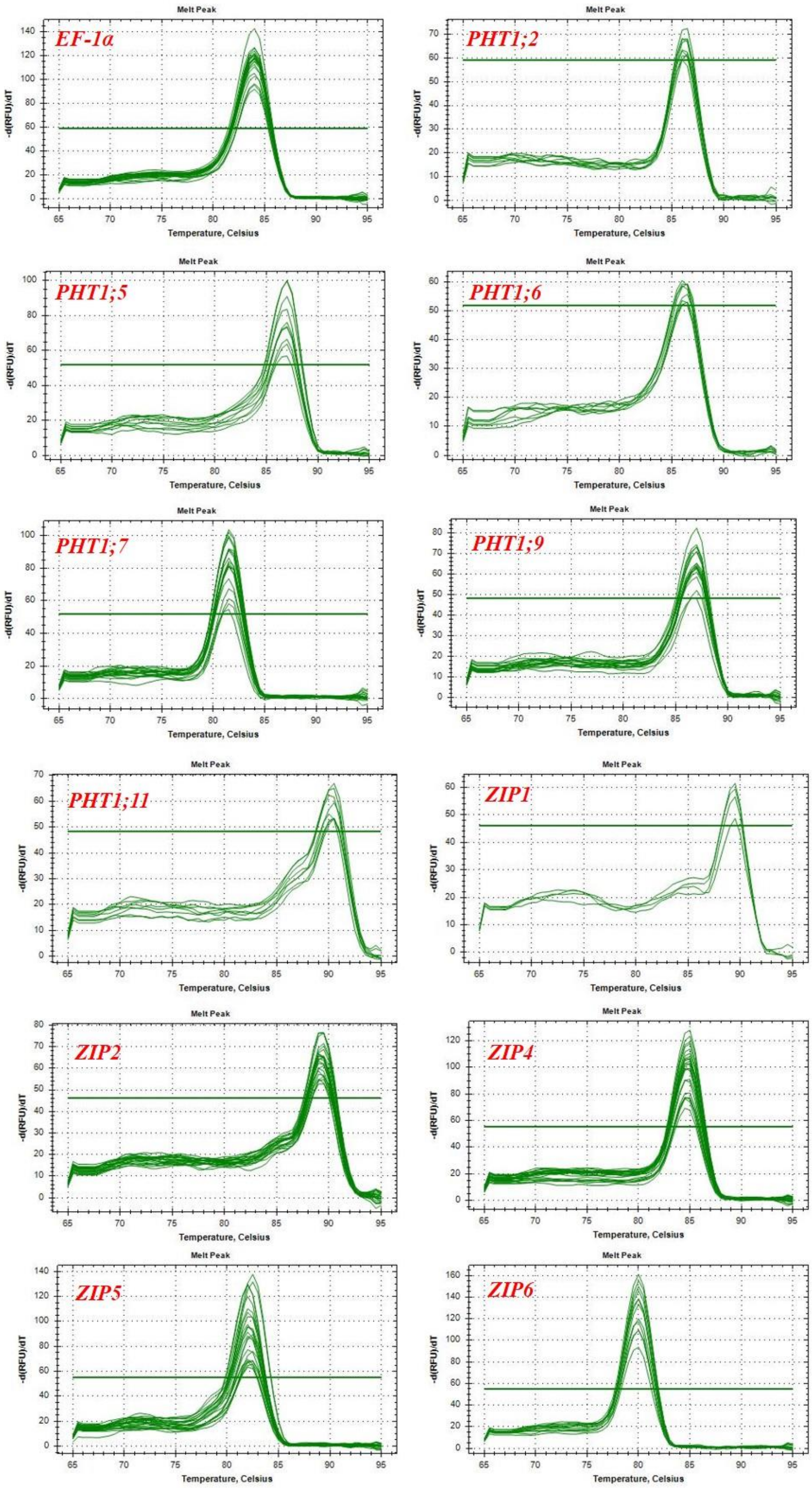

Figure S1. Images of melt curves of *EcEF-1α*, *EcPHT1,* and *EcZIP* genes in Quantitative real-time RT-PCR (qRT-PCR) analysis for all samples. The number of peaks indicates the number of tissues having that particular gene expressed. The height of the melt peak indicates the corresponding melting temperature of DNA.
